# The Capacitive Property Enhancement of CoFeP-Ni(OH)_2_/Nickel Foam Electrodes via an Interfacial Integration Strategy for Asymmetric Supercapacitors

**DOI:** 10.3390/molecules30142986

**Published:** 2025-07-16

**Authors:** Meiying Cui, Meiying Pei, Seok Kim

**Affiliations:** 1 School of Chemical Engineering, Pusan National University, Busandaehak-ro 63-2 beon-gil, Geumjeong-gu, Busan 46241, Republic of Korea; cuimy1997@163.com (M.C.); pmy0230@163.com (M.P.); 2 Institute of Environment and Energy, 2, Busandaehak-ro 63 beon-gil, Geumjung-gu, Busan 46241, Republic of Korea

**Keywords:** phosphide, transition metal compounds, pseudocapacitor, asymmetric supercapacitors, electrodeposition

## Abstract

We report the fabrication of CoFeP-Ni(OH)_2_/nickel foam (NF) composite electrodes via a two-step strategy involving the hydrothermal synthesis of Ni(OH)_2_ on nickel foam followed by the electrochemical deposition of CoFeP. The integration of the Ni(OH)_2_ interlayer not only provides a structurally robust interface but also facilitates synergistic redox activity, thereby significantly boosting the pseudocapacitive behavior of the electrode. Comparative analysis with bare CoFeP/NF reveals that the presence of the Ni(OH)_2_ layer contributes to enhanced charge transfer efficiency and an increased electroactive surface area. Among the samples prepared under varying deposition cycles, the optimized CoFeP-Ni(OH)_2_/NF electrode exhibits a high areal capacitance of 4244 mF cm^−2^ at 2 mA cm^−2^. Furthermore, an asymmetric supercapacitor device assembled with CoFeP-Ni(OH)_2_/NF as the positive electrode and activated carbon as the negative electrode delivers a maximum energy density of 0.19 mWh cm^−2^ at a power density of 0.37 mW cm^−2^ and excellent cycling stability, retaining 72% of its initial capacitance after 5000 cycles at a high current density of 8 mA cm^−2^.

## 1. Introduction

As the need for efficient energy storage in portable electronics and electric vehicles continues to rise, supercapacitors have garnered significant interest owing to their excellent power density, fast charge–discharge rates, and exceptional cycling stability [1,2,3]. Among the various types, asymmetric supercapacitors (ASCs) have emerged as promising candidates by effectively extending the operating voltage and enhancing energy density through the strategic combination of different electrode materials [4,5,6]. To fully realize the potential of ASCs, the design and engineering of electrode materials are of paramount importance. In particular, developing positive electrode materials with high capacitance, structural stability, and good conductivity is essential to achieving high energy density and long-term cycling durability [7,8,9]. Transition metal-based compounds, such as hydroxides and phosphides, have attracted growing interest due to their abundant redox activity, tunable electronic structure, and diverse chemical states. However, issues such as poor intrinsic conductivity or limited electroactive site exposure in some single-phase materials still restrict their full utilization [10,11,12,13]. To address these limitations, the construction of hybrid or hetero-structured electrodes that integrate complementary functionalities has emerged as an effective strategy, enabling the synergistic enhancement of electrochemical performance.

Transition metal hydroxides have attracted significant attention due to their excellent stability and high specific capacitance. Among them, Ni(OH)_2_ nanosheets have been extensively employed as effective interfacial modifiers and structural precursors owing to their layered architecture, high surface area, and abundant redox-active sites [14]. Their unique two-dimensional morphology facilitates efficient electron/ion transport and provides a favorable platform for the subsequent growth of functional materials, making them ideal candidates for constructing advanced composite electrodes [15,16]. Abd-Elrahim et al. fabricated a hybrid electrode through a single-step electrodeposition process, integrating CoS_X_–graphene nanoplatelets onto Ni(OH)_2_ nanosheets. In this configuration, the Ni(OH)_2_ substrate functioned as both a structural framework and an electrochemically active template, contributing to enhanced charge storage. The optimized heterostructure exhibited a high specific capacitance of 1618 F g^−1^ (1 A g^−1^), highlighting the synergistic interaction between the CoS_X_–graphene composite and the Ni(OH)_2_ support [17]. In a related study, Li et al. engineered a PPy-decorated ZnCo_2_O_4_@ Ni(OH)_2_ electrode via combined hydrothermal synthesis and polymerization. Here, the Ni(OH)_2_ nanosheets provided a conductive, porous interface that facilitated electron transfer and structural stability for the ZnCo_2_O_4_ and PPy components. This hierarchical design achieved an exceptional areal capacitance of 1256 C g^−1^ (1 A g^−1^) while retaining 81% of its initial capacity after 10,000 cycles, underscoring the pivotal role of Ni(OH)_2_ in promoting interfacial charge transport and long-term cycling performance [18].

Transition metal-based phosphides have recently garnered attention as promising pseudocapacitive materials due to their high electrical conductivity and multiple redox-active sites [12,19,20]. Monometallic phosphides such as CoP and NiP often suffer from limited electrochemical stability and the insufficient utilization of active sites during cycling [21,22]. In contrast, bimetallic or multimetallic phosphides, such as NiCoP or CoNiMn-P, can offer enhanced performance due to synergistic interactions between different metal species, which facilitate improved charge transport and increased redox-active sites, leading to enhanced electrochemical performance [23,24]. Wang et al. developed a ternary transition metal phosphide electrode (CoNiMn-P) using metal–organic framework precursors, achieving an outstanding specific capacitance of 2247 F g^−1^ at 1 A g^−1^. In practical applications, the assembled hybrid supercapacitor device exhibited superior energy storage performance, reaching an energy density of 45.7 Wh kg^−1^ (344.8 W kg^−1^) while maintaining 84.3% capacitance retention after 10,000 cycles [25]. Similarly, Anuratha et al. fabricated NiCoP electrodes through potentiostatic electrodeposition, demonstrating exceptional cycling stability with 86.7% capacity retention over 10,000 cycles [26]. The electrodeposition technique provides distinct advantages for electrode fabrication, enabling precise control over material composition, microstructure, and morphology through the adjustment of deposition parameters (current density, duration, and electrolyte formulation) [27,28].

In this study, we present a rationally designed hybrid electrode composed of bimetallic phosphide (CoFeP) nanoparticles anchored onto Ni(OH)_2_ nanosheets, which were directly grown on nickel foam through a two-step process involving hydrothermal synthesis and subsequent electrodeposition. The Ni(OH)_2_ nanosheets serve not only as a structural scaffold but also as a redox-active support, enhancing interfacial synergy and promoting the uniform growth of CoFeP. This binder-free configuration facilitates efficient charge transport and increases electroactive site exposure. The hierarchical integration of hydrothermally grown Ni(OH)_2_ nanosheets with electrodeposited CoFeP nanoparticles creates an efficient conductive network that enhances both pseudocapacitive performance and structural integrity. Furthermore, the practical viability of this design is confirmed through the assembly of an asymmetric supercapacitor device CoFeP- Ni(OH)_2_/NF//AC, exhibiting exceptional energy storage characteristics (0.19 mWh cm^−2^ at 0.37 mW cm^−2^) and cycling durability (72% retention after 5000 cycles) at a high current density of 8 mA cm^−2^.

## 2. Results and Discussion

### 2.1. Material Characterization

**Scheme 1:**
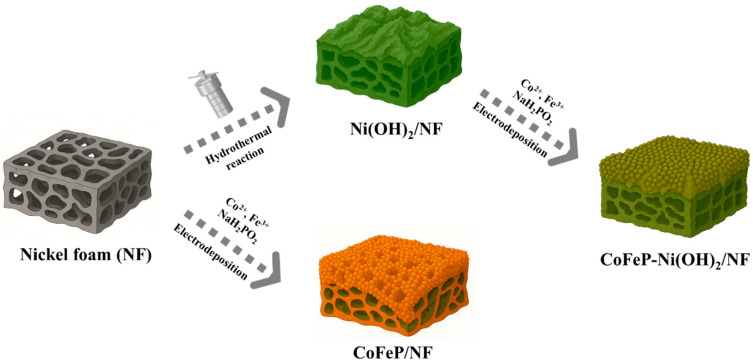
A schematic illustration of the fabrication process for CoFeP-Ni(OH)_2_/NF electrodes.

**Figure 1:**
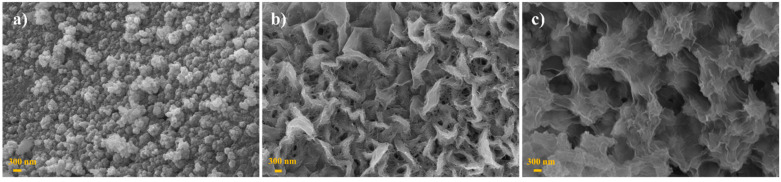
FE-SEM images of (**a**) CoFeP/NF, (**b**) Ni(OH)_2_/NF, and (**c**) CoFeP-Ni(OH)_2_/NF electrodes.

The CoFeP/NF, Ni(OH)_2_/NF, and CoFeP-Ni(OH)_2_/NF electrodes were fabricated according to the synthesis route illustrated in Scheme 1. Ni(OH)_2_/NF was first prepared via a hydrothermal method, followed by the electrodeposition of CoFeP onto its surface to form the CoFeP-Ni(OH)_2_/NF composite. The surface morphologies of the as-prepared electrode materials were investigated by field-emission scanning electron microscopy (FE-SEM), as shown in Figure 1. The CoFeP/NF sample (Figure 1a) presents a dense aggregation of nanoparticles uniformly anchored on the surface of nickel foam, forming a granular structure that facilitates abundant electroactive sites. In comparison, the Ni(OH)_2_/NF sample (Figure 1b) presents a wrinkled and porous nanosheet structure, which is likely to facilitate ion transport and improve electrolyte accessibility. Remarkably, the CoFeP-Ni(OH)_2_/NF hybrid (Figure 1c) shows an interconnected, loosely stacked nanosheet structure with irregular folding, combining the advantages of both nanoparticle-rich and porous architectures. This synergistic integration provides a large surface area and improved accessibility for redox reactions, which are favorable for enhancing pseudocapacitive performance.

**Figure 2:**
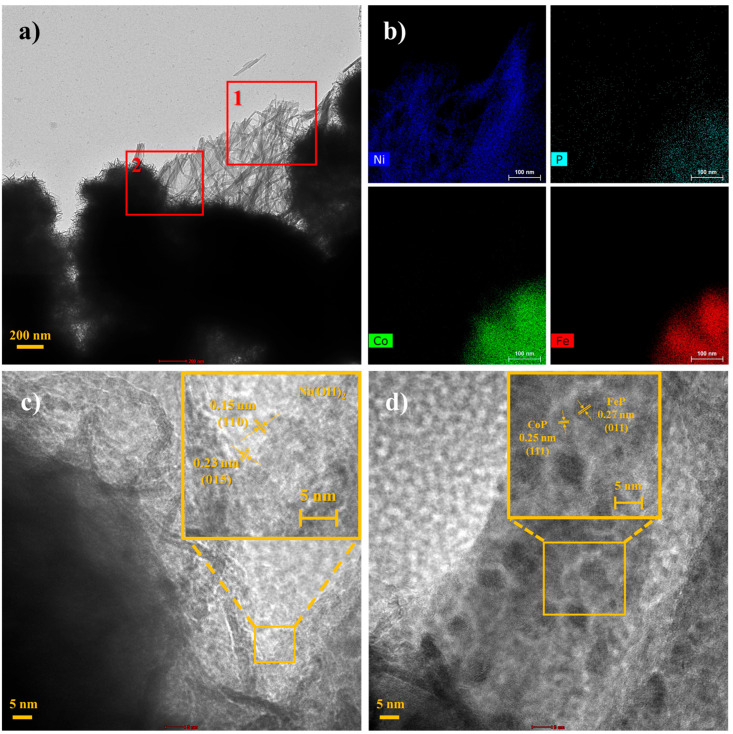
(**a**) A low-magnification TEM image of the CoFeP-Ni(OH)_2_/NF composite; (**b**) elemental mapping images of Ni, Co, Fe, and P corresponding to region 1 in (**a**,**c**); and (**d**) HR-TEM images from region 2 in (**a**).

Beyond surface-level observations, the internal structure and phase integration were further investigated using transmission electron microscopy (TEM), energy-dispersive X-ray spectroscopy (EDS), and high-resolution TEM (HR-TEM) analyses, as shown in Figure 2. The TEM image (Figure 2a) clearly reveals that CoFeP particles are intimately integrated with the nanosheet structure of Ni(OH)_2_, indicating the formation of a well-defined heterointerface between the two components. The particulate morphology corresponds to the CoFeP domains, while the thin, layered nanosheets are attributed to Ni(OH)_2_. This structural assignment is further corroborated by the EDS elemental mapping, which is shown in Figure 2b, where Co, Fe, and P signals are predominantly located in the particle regions, and Ni is uniformly distributed throughout the nanosheet matrix, confirming the successful spatial separation and combination of the two phases. HR-TEM analysis provides a direct insight into the crystallographic nature of each component. As shown in Figure 2c, the observed lattice spacings of 0.15 nm and 0.23 nm correspond to the (110) and (015) planes of Ni(OH)_2_, respectively, confirming the presence of crystalline hydroxide domains. In Figure 2d, distinct lattice fringes with interplanar distances of 0.25 nm and 0.27 nm were indexed to the (111) plane of CoP and the (011) plane of FeP, respectively, verifying the coexistence of CoP and FeP in the bimetallic phosphide phase.

**Figure 3:**
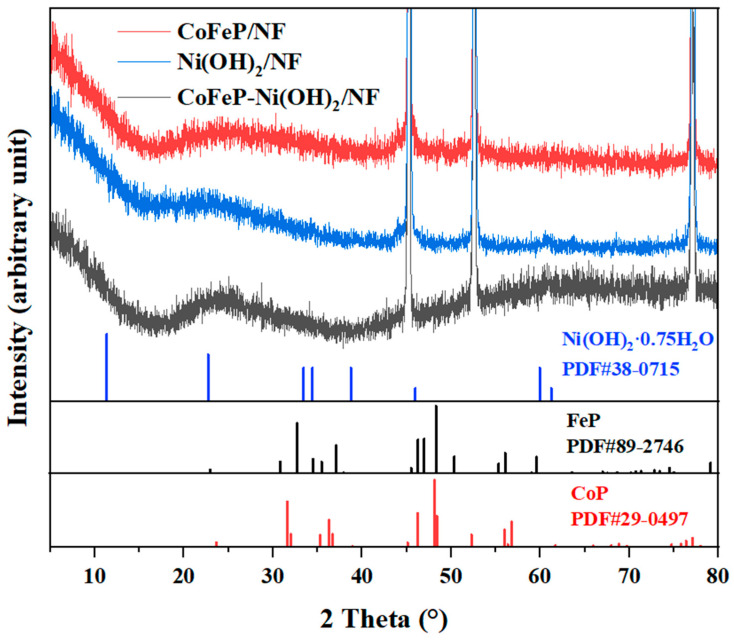
XRD patterns of CoFeP/NF, Ni(OH)_2_/NF, and CoFeP-Ni(OH)_2_/NF.

To confirm the phase composition and crystalline nature of the synthesized electrodes, X-ray diffraction (XRD) analysis was subsequently performed, as shown in Figure 3. In Figure 3, the XRD patterns of CoFeP/NF, Ni(OH)_2_/NF, and CoFeP–Ni(OH)_2_/NF appear weak because the main diffraction peaks at approximately 45°, 52°, and 77° correspond to the underlying Ni foam substrate.

The CoFeP/NF sample exhibits broad peaks characteristic of amorphous or poorly crystalline transition metal phosphides, with weak diffraction signals corresponding to FeP (PDF#89-2746) and CoP (PDF#29-0497), indicating the formation of a bimetallic phosphide phase [29]. For the Ni(OH)_2_/NF sample, the diffraction pattern shows distinct peaks that match well with the standard pattern of Ni(OH)_2_·0.75H_2_O (PDF#38-0715), confirming the successful deposition of layered hydroxide. Notably, the XRD pattern of the CoFeP-Ni(OH)_2_/NF composite exhibits overlapping features of both Ni(OH)_2_ and CoFeP, indicating the coexistence of phosphide and hydroxide phases. Additionally, the relatively broad and low-intensity peaks suggest a partially amorphous structure, which is favorable for enhancing electrochemical performance due to the increase in active surface area and ion accessibility.

### 2.2. Electrochemical Analysis

To elucidate the role of interface integration and the synergistic effect in CoFeP-Ni(OH)_2_/NF, the electrochemical performances of the three electrode configurations CoFeP/NF, Ni(OH)_2_/NF, and CoFeP-Ni(OH)_2_/NF were systematically compared.

First of all, to investigate the influence of the deposition cycle number on the electrochemical properties of the CoFeP-Ni(OH)_2_/NF electrode, samples were synthesized with 15, 18, 21, and 24 cycles of electrodeposition, and their performances were systematically compared. As shown in Figure S1a,b (Supplementary Materials), the CV and GCD curves clearly demonstrate that the electrochemical response intensifies with increasing cycle numbers up to 21 cycles, after which it declines at 24 cycles. Specifically, the areal capacitance increases from 2250 mF cm^−2^ (15 cycles) to a maximum of 3150 mF cm^−2^ (21 cycles), followed by a drop to 2260 mF cm^−2^ at 24 cycles, as depicted in Figure S1c. This trend suggests that moderate deposition allows for optimal active material coverage and efficient ion diffusion pathways, whereas excessive deposition beyond the critical point (21 cycles) may result in overloading, increased internal resistance, and reduced electrochemical accessibility. The EIS spectra in Figure S1d further support this conclusion. The sample with 21 deposition cycles exhibits the smallest charge transfer resistance (R_ct_), exhibiting the most favorable electron transport kinetics among the group.

**Figure 4:**
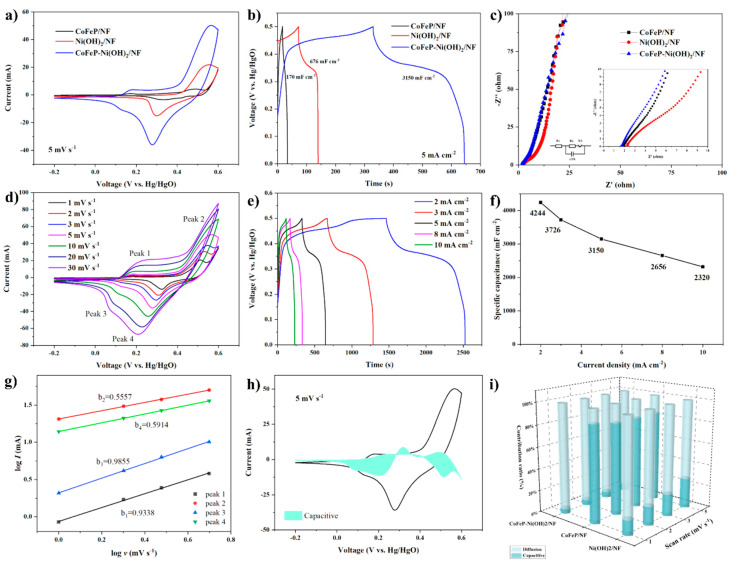
Electrochemical performance comparison of CoFeP/NF, Ni(OH)_2_/NF, and CoFeP-Ni(OH)_2_/NF electrodes: (**a**) CV curves at 5 mV s^−1^; (**b**) GCD curves at 5 mA cm^−2^; (**c**) Nyquist plots with the inset of a high-frequency region; (**d**) CV curves of CoFeP-Ni(OH)_2_/NF at various scan rates; (**e**) corresponding GCD curves at different current densities; (**f**) areal capacitance versus current density; (**g**) b-value analysis of peaks in (**b**); (**h**) capacitive contribution analysis at 5 mV s^−1^; and (**i**) a comparison of capacitive contribution among the three electrodes at multiple current densities.

As shown in Figure 4a,b, the sample with 21 deposition cycles is referred to as CoFeP-Ni(OH)_2_/NF and exhibits a significantly larger CV area and longer GCD time than the other two electrodes, indicating an enhanced charge storage capacity. The areal capacitances of CoFeP/NF, Ni(OH)_2_/NF, and CoFeP-Ni(OH)_2_/NF under a current density of 5 mA cm^−2^, calculated using Equation (1), are 170 mF cm^−2^, 676 mF cm^−2^, and 3150 mF cm^−2^, respectively. Notably, the CoFeP-Ni(OH)_2_/NF electrode demonstrates capacitance values approximately 18.5 and 5 times higher than those of CoFeP/NF and Ni(OH)_2_/NF, respectively. This enhancement can be attributed to the structural synergy between the redox-active Ni(OH)_2_ interface layer and the outer CoFeP deposition, which enables efficient ion diffusion and improved electron transport pathways. Nyquist plots in Figure 4c further reveal that CoFeP-Ni(OH)_2_/NF possesses a smaller R_ct_, suggesting better conductivity and faster interfacial kinetics [30,31]. Figure 4d presents the CV curves of the CoFeP-Ni(OH)_2_/NF electrode recorded at various scan rates. Two distinct pairs of redox peaks can be observed, where peaks 1 and 2 correspond to oxidation processes, while peaks 3 and 4 are attributed to reduction reactions. As the scan rate increases, oxidation peaks gradually shift to more positive potentials, while the reduction peaks move toward more negative potentials. This phenomenon is attributed to increased electrode polarization at higher scan rates, which hampers the redox reaction kinetics and results in larger overpotentials [32,33]. In Figure 4e, GCD curves at various current densities are shown, and the corresponding areal-specific capacitances were calculated using Equation (1), as shown in Figure 4f. The capacitance decreases from 4494 to 2320 mF cm^−2^ as the current density increases from 1 to 10 mA cm^−2^. Despite this decrease, the electrode retains over 51.6% of its initial capacitance, indicating excellent capability rates and the presence of highly accessible ion diffusion pathways under high current conditions.

(1) $${Cs}_{A}=\frac{I\times t}{S\times ∆V}$$

Here, I, t, S, and ∆V, in order, refer to the current (mA), the discharging time (s), the area loading of active material (cm^−2^), and the potential window (V).

To further elucidate the charge storage mechanism of the CoFeP-Ni(OH)_2_/NF electrode, the b-values for multiple redox peaks were extracted from the log(*I*)–log(*v*) plots and are shown in Figure 4g based on the following Formula (2):

*I* = *aν^b^* (2)

where *I* is the measured current, *v* is the scan rate, and a and b are the adjustable constant. The fitted b-values for peaks 1 to 4, as shown in Figure 4g, are 0.9338, 0.5557, 0.9855, and 0.5914, respectively. These values, ranging between 0.5 and 1.0, suggest a combination of diffusion-controlled and surface-controlled charge storage processes. Notably, peaks 1 and 3 are closer to 1.0, indicating capacitive-dominated behavior, whereas peaks 2 and 4, with b-values nearer to 0.5, reflect a stronger diffusion-controlled contribution. This implies that distinct redox processes within the electrode respond differently to the scan rate, reflecting a kinetically mixed but highly reversible charge storage mechanism.

Figure 4h presents the CV curve of the CoFeP-Ni(OH)_2_/NF electrode at a scan rate of 5 mV s^−1^, where the shaded region represents the capacitive contribution. The capacitive and diffusion-controlled portions at other scan rates are provided in Figure S2. The capacitive contribution was quantitatively determined based on the following Equation (3):

*i*(*V*) = *k*_1_*v* + *k*_2_*v*^1/2^ (3)

where *i* and *v* represent the measured current and sweep rate, respectively. *k_1_* corresponds to the capacitive (surface-controlled) contribution, and k_2_ is related to diffusion-controlled processes. This method enables the deconvolution of the total current into capacitive and diffusion-limited components, providing further insight into the charge storage mechanism. The capacitive contributions of CoFeP/NF, Ni(OH)_2_/NF, and CoFeP-Ni(OH)_2_/NF electrodes are summarized in Figure 4i. It is evident that CoFeP/NF exhibits a higher capacitive contribution, indicating a surface-controlled charge storage behavior. In contrast, both Ni(OH)_2_/NF and CoFeP-Ni(OH)_2_/NF display lower capacitive ratios, suggesting that their charge storage is predominantly governed by diffusion-controlled processes. The capacitive contributions of CoFeP-Ni(OH)_2_/NF, ranging from 4.4% to 6.2%, are significantly lower than those of CoFeP/NF (83%) and Ni(OH)_2_/NF (14%), indicating that the charge storage behavior is predominantly governed by battery-type diffusion-controlled processes after integration [34]. After the integration of CoFeP and Ni(OH)_2_, the electrode exhibits a lower capacitive contribution and a higher proportion of diffusion-controlled behavior. This may be attributed to the complex interfacial structure and the introduction of mixed oxygen species. Different types of oxygen species, such as lattice oxygen, surface hydroxyl groups, and chemisorbed oxygen, can significantly influence the pseudocapacitive behavior of transition metal-based electrodes. The presence of Ni(OH)_2_ and CoFeP components is expected to introduce mixed oxygen species that contribute to redox activity and charge storage [35].

**Figure 5:**
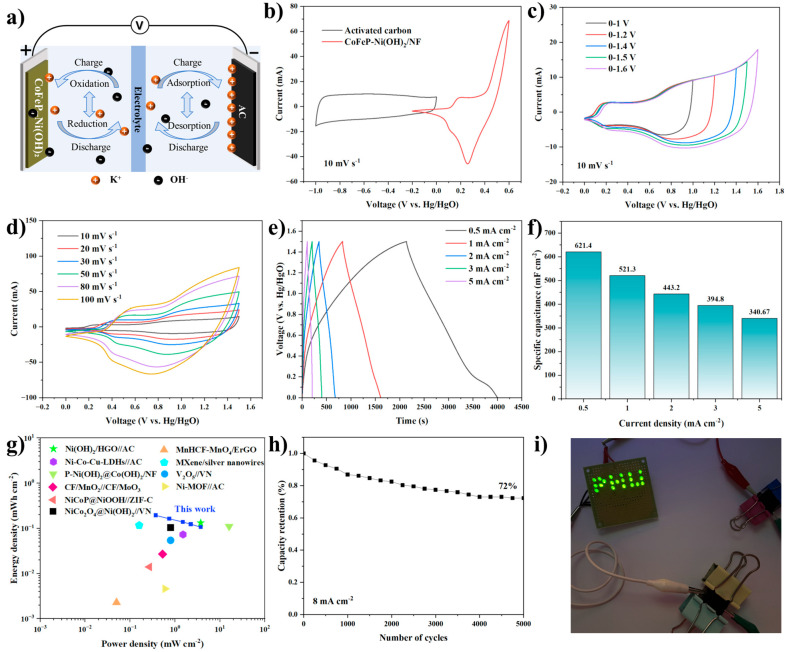
(**a**) A schematic illustration of the CoFeP-Ni(OH)_2_/NF//AC ASC device; (**b**) CV curves of the CoFeP-Ni(OH)_2_/NF electrodes and AC electrodes at a scan rate of 10 mV s^−1^; (**c**) CV curves of the ASC at different voltage windows; (**d**) CV curves of the ASC at various scan rates in a 0-1.5V voltage window; (**e**) GCD curves of the ASC at different current densities; (**f**) areal specific capacitance calculated at various current densities; (**g**) a Ragone plot comparing energy and power density with previously reported ASCs; (**h**) cycling performance at 8 mA cm^−2^ over 5000 cycles; and (**i**) the optical image of a powered LED panel driven by the assembled ASC device.

**Figure 6:**
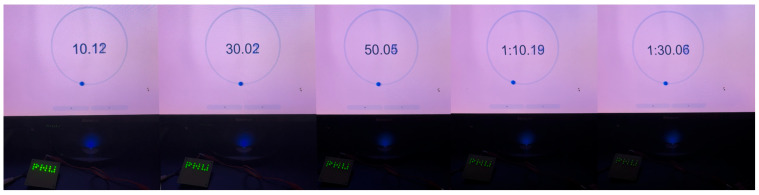
A photographic demonstration of the LED panel (composed of 20 LEDs and displaying the letters “PNU”) powered by the asymmetric supercapacitor device over time.

For further investigation, an asymmetric supercapacitor (ASC) device was assembled using CoFeP-Ni(OH)_2_/NF as the positive electrode, activated carbon (AC) as the negative electrode, and PVA/KOH gel as the electrolyte. A schematic illustration of the device configuration is shown in Figure 5a. As shown in Figure 5b, the CV curves of CoFeP-Ni(OH)_2_/NF (−0.2 V~0.6 V) and AC (−1 V~0 V) electrodes at a scan rate of 10 mV s^−1^ were first recorded individually. The assembled ASC device exhibited a typical capacitive response, integrating the Faradaic redox behavior of the positive electrode with the electrical double-layer characteristics of the AC electrode. To determine the optimal operating voltage, CV curves were recorded at various potential windows from 1 V to 1.6 V, as depicted in Figure 5c. The device maintained a stable shape without significant polarization to 1.5 V, confirming this as the appropriate voltage window for further analysis [36]. Figure 5d displays the CV profiles at scan rates ranging from 10 to 100 mV s^−1^ exhibiting a quasi-rectangular shape with redox peaks, which is indicative of combined pseudocapacitive and EDLC-type behavior. The shape was well-retained at higher scan rates, implying excellent capability rates and fast charge transfer kinetics. GCD curves at different current densities are shown in Figure 5e, and the areal-specific capacitance is summarized in Figure 5f, which was calculated using Equation (1). The device delivers a maximum capacitance of 621.4 mF cm^−2^ at 0.5 mA cm^−2^ and maintains 340.67 mF cm^−2^ even at a high current density of 5 mA cm^−2^, corresponding to a capacitance retention of 54.8%, which demonstrates a good rate of capability. The practical electrochemical performance of the CoFeP-Ni(OH)_2_/NF//AC ASC device was further evaluated. As shown in Figure 5g, the Ragone plot demonstrates a favorable balance between areal energy and power densities, with the device achieving a maximum energy density of 0.19 mWh cm^−2^ at a power density of 0.37 mW cm^−2^, as calculated using Equations (4) and (5). These values are competitive with or superior to those of previously reported transition metal-based systems, including Ni(OH)_2_/HGO//AC [37], MnHCF-MnO_4_/ErGO [38], Ni-Co-Cu-LDHs//AC- [39], MXene/silver nanowire [40], P-Ni(OH)_2_@Co(OH)_2_/NF [41], V_2_O_5_//VN [42], CF/MnO_2_//CF/MoO_3_ [43], CoNiP@NiOOH//ZIF-C [44], Ni-MOF//AC [45], and NiCo_2_O_4_@Ni(OH)_2_//VN [46] based devices, as summarized in Table S1. Long-term cycling stability was assessed at a high current density of 8 mA cm^−2^ over 5000 cycles (Figure 5h), with the device retaining 72% of its initial capacitance, indicating decent long-term cycling stability under harsh conditions. To confirm its applicability, the assembled device was used to power a 20-LED panel displaying the letters “PNU”, shown in Figure 5i. The lit panel remained continuously illuminated for over 90 min (Figure 6), demonstrating both the practical energy output and operational stability of the system.

(4) $$E=\frac{Cs\times V^{2}}{2\times 3600}$$

(5) $$P=\frac{E\times 3600}{t}$$

where *Cs*, *V*, and *t* are the specific capacitance (mF cm^−2^), the potential window (V), and the discharging time (s) of the hybrid supercapacitor, respectively.

## 3. Materials and Methods

### 3.1. Materials

The materials used were sodium dodecyl sulfate (SDS), nickel nitrate hexahydrate (Ni(NO_3_)_2_·6H_2_O), cobalt nitrate hexahydrate (Co(NO_3_)_2_·6H_2_O), ferric chloride hexahydrate (FeCl_3_·6H_2_O), urea, polyvinyl alcohol (PVA), potassium hydroxide (KOH), sodium sulfate (Na_2_SO_4_), sodium dihydrogen phosphate monohydrate (NaH_2_PO_2_·H_2_O), polyvinylidene fluoride (PVdF), N-methyl-2-pyrrolidone (NMP), carbon black, and hydrogen chloride (HCl).

### 3.2. Preparation of Ni(OH)_2_/NF

The nickel foam (NF) was pre-treated by first immersing it in acetone to remove surface oxides and impurities. Subsequently, it was cleaned with a 1:1 volume ratio of HCl and deionized (DI) water, followed by thorough rinsing with DI water and ethanol. Finally, the foam was dried completely before further use. A solution containing 0.5 mmol of Ni(NO_3_)_2_·6H_2_O, 3.5 mmol of urea, and 1.4 mmol of SDS was prepared in 50 mL of DI water and transferred into a Teflon-lined stainless-steel autoclave. Pre-treated Ni foam was then placed in the solution at an inclined angle, fully immersed but without direct contact with the bottom of the autoclave. The autoclave was sealed and maintained at 120 °C for 12 h. After the hydrothermal reaction, Ni foam was thoroughly rinsed with DI water and ethanol to remove residual reactants. The Ni(OH)_2_-coated Ni foam was named Ni(OH)_2_/NF.

### 3.3. Preparation of CoFeP/NF and CoFeP-Ni(OH)_2_/NF

The CoFeP-Ni(OH)_2_/NF electrodes were fabricated via an electrochemical deposition process using the cyclic voltammetry (CV) method. The electrolyte consisted of 0.01 M Co(NO_3_)_2_·6H_2_O, 0.01 M FeCl_3_·6H_2_O, 0.02 M NaH_2_PO_2_·H_2_O, and 0.02 M Na_2_SO_4_. The Ni(OH)_2_/NF was used as the working electrode, a Pt wire served as the counter electrode, and an Ag/AgCl electrode was employed as the reference. Electrodeposition was carried out in the voltage range of −1.2 V to −0.5 V for 15, 18, 21, and 24 cycles, with the resulting samples designated as CoFeP-Ni(OH)_2_-15, CoFeP-Ni(OH)_2_-18, CoFeP-Ni(OH)_2_-21, and CoFeP-Ni(OH)_2_-24, respectively. Among these, the sample obtained after 21 cycles is referred to as CoFeP-Ni(OH)_2_/NF in the following discussions for simplicity. For comparison, CoFeP/NF was synthesized using the same electrodeposition method described above, with 21 CV cycles applied.

### 3.4. Preparation of PVA/KOH Electrolyte

Based on a previous study, solution A was prepared by dissolving 3 g of PVA in 30 mL of distilled water at 80 °C, and this was stirred for 2 h. Separately, solution B was prepared using 1.8 g of KOH dissolved in 5 mL of DI water. After solution A turned transparent, solution B was introduced, followed by continuous stirring for 30 min. The mixture was subsequently kept at room temperature to allow the formation of the PVA/KOH hydrogel [41].

### 3.5. Preparation of Activated Carbon (AC) Electrode

The AC electrode was prepared by mixing activated carbon, carbon black, and PVdF in a weight ratio of 8:1:1 using NMP as the solvent. The resulting slurry was uniformly coated onto a piece of pre-cleaned nickel foam and subsequently dried at 60 °C for 12 h under vacuum to remove the residual solvent, forming the AC electrode.

### 3.6. Materials Characterization

To investigate the structural and chemical characteristics of the prepared materials, X-ray diffraction (XRD) was employed to assess crystallinity. The morphological features, elemental composition, and spatial distribution were characterized using scanning electron microscopy (SEM, Carl Zeiss/Supra 40, Berlin, Germany), field emission transmission electron microscopy (FE-TEM, ThermoFisher Scientific/TALOS F200X, Waltham, MA, USA), and energy-dispersive X-ray spectroscopy (EDS, ThermoFisher Scientific/TALOS F200X, Waltham, MA, USA). These complementary techniques enabled a detailed understanding of the physical and chemical nature of the synthesized samples.

### 3.7. Electrochemical Properties

The electrochemical behavior of the electrode materials and their device-level performance were evaluated through cyclic voltammetry (CV), galvanostatic charge–discharge (GCD), and electrochemical impedance spectroscopy (EIS), conducted using an Iviumstat electrochemical workstation (IviumSoft 4.1127, Ivium Technologies, Eindhoven, The Netherlands). The electrodes were carried out in a conventional three-electrode configuration, with the synthesized electrode as the working electrode, the Hg/HgO electrode as the reference, and a platinum wire serving as the counter electrode. The electrolyte used was 1 M KOH aqueous solution. For the assembly of a solid-state asymmetric supercapacitor, a PVA/KOH gel was employed as the ionic medium. The areal-specific capacitance (*C_SA_*, mA cm^−2^) was determined from the GCD profiles using Equation (1).

The asymmetric supercapacitor device was fabricated with CoFeP-Ni(OH)_2_/NF as the positive electrode, activated carbon (AC) as the negative electrode, and the PVA/KOH used for the electrolyte. To ensure charge balance between the two electrodes, the area loading on a negative electrode of AC was balanced using Equation (6) below:

(6) $$S_{+}\;\times \;{Cs}_{+}\;\times \;{∆V}_{+}\;=\;S_{-}\;\times \;{Cs}_{-}\;\times \;{∆V}_{-}$$

where *S* is the area (cm^2^) of the material loading on the electrode, *Cs* is the specific capacitance (mF cm^−2^), ∆V is the potential window (V), and the + and − of their subscripts represent positive and negative electrodes.

The energy density (mWh cm^−2^) and power density (mW cm^−2^) of the hybrid supercapacitor were calculated using Equations (4) and (5).

## 4. Conclusions

In conclusion, we successfully developed a hybrid electrode by integrating CoFeP nanoparticles onto Ni(OH)_2_ nanosheets grown in situ on nickel foam through a two-step synthesis approach involving hydrothermal growth and electrochemical deposition. The incorporation of Ni(OH)_2_ served as a mechanically stable interfacial layer and redox-active scaffold, which facilitated improved charge transport and the increased exposure of active sites. Among the various deposition conditions tested, the electrode synthesized with 21 CV cycles exhibited superior electrochemical performance, achieving a high areal capacitance of 4244 mF cm^−2^ at 2 mA cm^−2^ and an excellent capability rate. When applied in an asymmetric supercapacitor configuration with activated carbon as the negative electrode, the device delivered a peak energy density of 0.19 mWh cm^−2^ at a power density of 0.37 mW cm^−2^ and maintained 72% of its initial capacitance after 5000 cycles at a high current. These findings demonstrate the effectiveness of combining transition metal hydroxides and bimetallic phosphides in a binder-free architecture, providing a promising route toward high-performance supercapacitor electrodes for next-generation energy storage applications.

## Data Availability

The raw data supporting the conclusions of this article will be made available by the authors on request.

## Supplementary Materials

The following supporting information can be downloaded at https://www.mdpi.com/article/10.3390/molecules30142986/s1. Figure S1: Electrochemical characterization of CoFeP-Ni(OH)2/NF electrodes synthesized with varying electrodeposition cycles (15, 18, 21, 24 cycles); Figure S2: CV of diffusion-controlled contributions of CoFeP-Ni(OH)2/NF. Table S1: Summary of the power and energy densities of various supercapacitor systems reported in previous studies [37,38,39,40,41,43,44,45,46,47].
