# Supplementary material for: The Capacitive Property Enhancement of CoFeP-Ni(OH)2/Nickel Foam Electrodes via an Interfacial Integration Strategy for Asymmetric Supercapacitors
Source: Molecules. 2025 Jul 16;30(14):2986. doi: 10.3390/molecules30142986 (PMC12298675; doi:10.3390/molecules30142986)
Supplement: Supplementary file 1 [file molecules-30-02986-s001.zip › molecules-3753279-supplementary.pdf]

*Supporting Information*

# The Capacitive Property Enhancement of CoFeP-$Ni(OH)_2$/Nickel Foam Electrodes via an Interfacial Integration Strategy for Asymmetric Supercapacitors

**Meiying Cui [1] [+], Meiying Pei [1] [+], Seok Kim [1,2] [*]**

[1] School of Chemical Engineering, Pusan National University, Busandaehak-ro 63-2 beon-gil, Geumjeong-gu, Busan 46241, South Korea

[2] Institute of Environment and Energy, 2, Busandaehak-ro 63 beon-gil, Geumjung-gu, Busan 46241, South Korea

\+ These authors contributed equally to this work

*corresponding author; seokkim@pusan.ac.kr

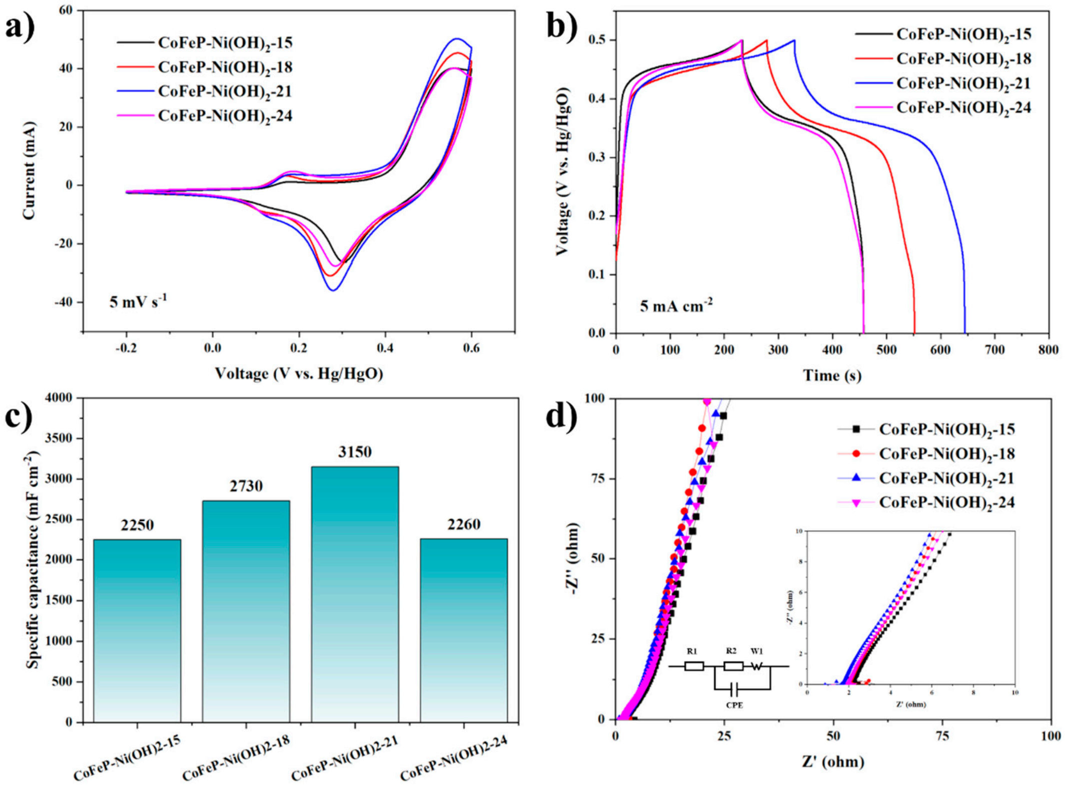


Figure S1. Electrochemical characterization of CoFeP-Ni(OH)$_2$/NF electrodes synthesized with varying electrodeposition cycles (15, 18, 21, 24 cycles): (a) Cyclic voltammetry (CV) curves at 5 mV s$^{-1}$; (b) Galvanostatic charge-discharge (GCD) profiles at 5 mA cm$^{-2}$; (c) Areal specific capacitance comparison calculated from GCD curves (b); (d) Electrochemical impedance spectroscopy (EIS) plots with the corresponding inset showing the high-frequency region.

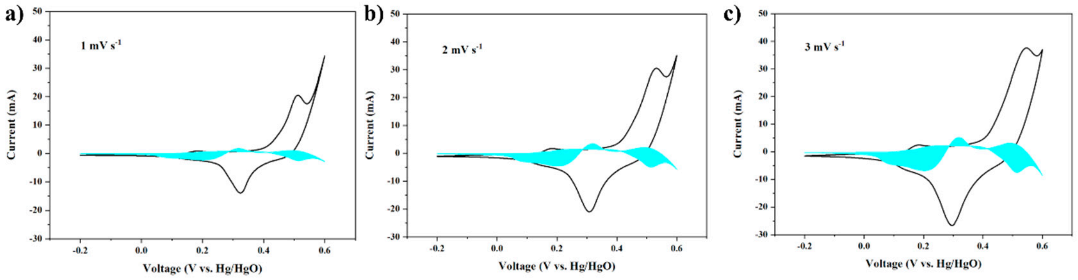


Figure S2. CV curves of the CoFeP-$Ni(OH)_2$/NF electrode at different scan rates (1, 2, and 3 mV $s^{-1}$), highlighting the capacitive (shaded areas) and diffusion-controlled contributions.

Table S1. Summary of the power and energy densities of various supercapacitor systems reported in previous studies.

| Type of SCs | Power density (mW $cm^{-2}$) | Energy density (mWh $cm^{-2}$) | Ref. |
|---|---|---|---|
| **$Ni(OH)_2$/HGO//AC** | 3.722 | 0.1339 | [1] |
| **Ni-Co-Cu-LDHs//AC** | 1.51 | 0.0734 | [2] |
| **P-$Ni(OH)_2$@$Co(OH)_2$/NF** | 16 | 0.11 | [3] |
| **MnHCF-$MnO_4$/ErGO** | 0.05 | 0.0023 | [4] |
| **MXene/silvernanowires** | 0.16 | 0.117 | [5] |
| **$V_2O_5$//VN** | 0.8014 | 0.0543 | [6] |
| **CoNiP@NiOOH // ZIF-C** | 0.53 | 0.027 | [7] |
| **CF/$MnO_2$ // CF/$MoO_3$** | 0.27 | 0.014 | [8] |
| **$NiCo_2O_4$@$Ni(OH)_2$ // VN** | 0.6 | 0.0046 | [9] |
| **Ni-MOF // AC** | 0.8 | 0.1038 | [10] |
| **CoFeP–$Ni(OH)_2$//AC** | 0.375 | 0.1942 | This work |

## Reference


1. Wu, X.; Zeng, F.; Song, X.; Sha, X.; Zhou, H.; Zhang, X.; Liu, Z.; Yu, M.; Jiang, C. In-situ growth of $Ni(OH)_2$ nanoplates on highly oxidized graphene for all-solid-state flexible supercapacitors. *Chem. Eng. J.* **2023**, *456*, 140947. https://www.sciencedirect.com/science/article/pii/S1385894722064270.
2. Sekhar, S.C.; Nagaraju, G.; Ramulu, B.; Arbaz, S.J.; Narsimulu, D.; Hussain, S.K.; Yu, J.S. An eco-friendly hot-water therapy towards ternary layered double hydroxides laminated flexible fabrics for wearable supercapatteries. *Nano Energy* **2020**, *76*, 105016. https://www.sciencedirect.com/science/article/pii/S2211285520305930.
3. Li, K.; Zhao, B.; Bai, J.; Ma, H.; Fang, Z.; Zhu, X.; Sun, Y. A High-Energy-Density Hybrid Supercapacitor with P-$Ni(OH)_2$@ $Co(OH)_2$ Core–Shell Heterostructure and $Fe_2O_3$ Nanoneedle Arrays as Advanced Integrated Electrodes. *Small* **2020**, *16*, 2001974. https://onlinelibrary.wiley.com/doi/full/10.1002/smll.202001974.
4. Liang, J.; Tian, B.; Li, S.; Jiang, C.; Wu, W. All-printed MnHCF-MnOx-based high-performance flexible supercapacitors. *Adv. Energy Mater.* **2020**, *10*, 2000022. https://advanced.onlinelibrary.wiley.com/doi/full/10.1002/aenm.202000022.
5. Cao, Z.; Liang, G.; Ho, D.; Zhi, C.; Hu, H. Interlayer Injection of Low-Valence Zn Atoms to Activate MXene-Based Micro-Redox Capacitors With Battery-Type Voltage Plateaus. *Adv. Funct. Mater.* **2023**, *33*, 2303060. https://advanced.onlinelibrary.wiley.com/doi/full/10.1002/adfm.202303060.
6. Zhao, J.; Lu, H.; Zhang, Y.; Yu, S.; Malyi, O.I.; Zhao, X.; Wang, L.; Wang, H.; Peng, J.; Li, X. Direct coherent multi-ink printing of fabric supercapacitors. *Sci. Adv.* **2021**, *7*, eabd6978. https://www.science.org/doi/full/10.1126/sciadv.abd6978.
7. Noh, J.; Yoon, C.-M.; Kim, Y.K.; Jang, J. High performance asymmetric supercapacitor twisted from carbon fiber/$MnO_2$ and carbon fiber/$MoO_3$. *Carbon* **2017**, *116*, 470–478. https://doi.org/10.1016/j.carbon.2017.02.033.
8. Qiu, M.; Sun, P.; Cui, G.; Tong, Y.; Mai, W. A flexible microsupercapacitor with integral photocatalytic fuel cell for self-charging. *ACS nano* **2019**, *13*, 8246–8255. https://pubs.acs.org/doi/10.1021/acsnano.9b03603.
9. Liu, L.; Feng, Y.; Liang, J.; Li, S.; Tian, B.; Yao, W.; Wu, W. Structure-designed fabrication of all-printed flexible in-plane solid-state supercapacitors for wearable electronics. *J. Power Sources* **2019**, *425*, 195–203. https://doi.org/10.1016/j.jpowsour.2019.03.118.
10. Yan, Y.; Gu, P.; Zheng, S.; Zheng, M.; Pang, H.; Xue, H. Facile synthesis of an accordion-like Ni-MOF superstructure for high-performance flexible supercapacitors. *J. Mater. Chem. A* **2016**, *4*, 19078–19085. https://doi.org/10.1039/C6TA08331E.
